# Measures of Listening Effort Are Multidimensional

**DOI:** 10.1097/AUD.0000000000000697

**Published:** 2019-08-23

**Authors:** Sara Alhanbali, Piers Dawes, Rebecca E. Millman, Kevin J. Munro

**Affiliations:** 1 Manchester Centre for Audiology and Deafness, School of Health Sciences, University of Manchester, Manchester, United Kingdom; 2 Manchester University Hospitals NHS Foundation Trust, Central Manchester University Hospitals National Health Service Foundation Trust, Manchester Academic Health Science Centre, Manchester, United Kingdom.

**Keywords:** Alpha power, Listening effort, Pupil size, Reaction time, Self-reported listening effort, Skin conductance

## Abstract

Listening effort can be defined as the cognitive resources required to perform a listening task. The literature on listening effort is as confusing as it is voluminous: measures of listening effort rarely correlate with each other and sometimes result in contradictory findings. Here, we directly compared simultaneously recorded multimodal measures of listening effort. After establishing the reliability of the measures, we investigated validity by quantifying correlations between measures and then grouping-related measures through factor analysis.

**Design:**

One hundred and sixteen participants with audiometric thresholds ranging from normal to severe hearing loss took part in the study (age range: 55 to 85 years old, 50.3% male). We simultaneously measured pupil size, electroencephalographic alpha power, skin conductance, and self-report listening effort. One self-report measure of fatigue was also included. The signal to noise ratio (SNR) was adjusted at 71% criterion performance using sequences of 3 digits. The main listening task involved correct recall of a random digit from a sequence of six presented at a SNR where performance was around 82 to 93%. Test–retest reliability of the measures was established by retesting 30 participants 7 days after the initial session.

**Results:**

With the exception of skin conductance and the self-report measure of fatigue, interclass correlation coefficients (ICC) revealed good test–retest reliability (minimum ICC: 0.71). Weak or nonsignificant correlations were identified between measures. Factor analysis, using only the reliable measures, revealed four underlying dimensions: factor 1 included SNR, hearing level, baseline alpha power, and performance accuracy; factor 2 included pupillometry; factor 3 included alpha power (during speech presentation and during retention); factor 4 included self-reported listening effort and baseline alpha power.

**Conclusions:**

The good ICC suggests that poor test reliability is not the reason for the lack of correlation between measures. We have demonstrated that measures traditionally used as indicators of listening effort tap into multiple underlying dimensions. We therefore propose that there is no “gold standard” measure of listening effort and that different measures of listening effort should not be used interchangeably. When choosing method(s) to measure listening effort, the nature of the task and aspects of increased listening demands that are of interest should be taken into account. The findings of this study provide a framework for understanding and interpreting listening effort measures.

## INTRODUCTION

In simple terms, listening effort has been defined as, “The mental exertion [effort] required to attend to, and to understand, an auditory message [listening]” (McGarrigle et al. 2016). Pichora-Fuller et al. (2016) provided a general definition of effort as, “The deliberate allocation of mental resources to overcome obstacles in goal pursuit when carrying out a [listening] task.” The “deliberate allocation” can be traced back to the limited capacity model (Kahneman 1973), where the individual can consciously choose where to allocate resources; however, there may be situations where there is involuntary use of cognitive resources that may not be amendable to conscious awareness (Strauss & Francis 2017). It has been traditionally assumed that the experience of listening effort is predominantly influenced by the demands of the listening task. However, recent interpretations of the concept of listening effort and its underlying mechanisms suggest that multiple dimensions influence the experience of listening effort (Pichora-Fuller et al. 2016; Peelle 2017; Strauss & Francis 2017). The deliberate allocation of cognitive resources (i.e., the amount of neural activity that is potentially available to an individual for information storing and processing [Grabner et al. 2010]) required to justify sustained effort is influenced by the motivation and reward associated with perceived performance. Therefore, Strauss and Francis (2017) suggested that in demanding listening tasks, it is not possible to assume that the amount of effort required to complete the task (demanded effort) equals the amount of effort that individuals actually exert (exerted effort). The effort that is applied by an individual to complete a task is a combination of (1) the demands of the task, (2) the cognitive resources, and (3) the motivation to use the cognitive resources. The influence of multiple factors on the experience of listening effort suggests that listening effort might be a multidimensional process. In support for the multidimensionality of listening effort, Peelle (2017) suggests that multiple cognitive systems are activated during effortful listening.

Individuals with hearing impairment experience increased listening effort despite using hearing aids or cochlear implants (Hornsby 2013; Alhanbali et al. 2017). People who experience high levels of “ineffective” listening effort are likely to report increased negative impacts on the social and emotional aspects of their life (Alhanbali et al. 2018). Sustained listening effort does not always result in perceived successful performance; in such cases, fatigue might develop as an adaptive state to halt the exertion of further effort (Hockey 2013; Alhanbali et al. 2018). Listening-related fatigue has been defined as “extreme tiredness resulting from [unrewarding] effortful listening” (McGarrigle et al. 2014). Identifying reliable clinical measures of listening effort may provide a means of indexing an important dimension of hearing disability that is currently not well captured by current audiological measures, such as pure-tone and speech audiometry, or self-reported measures of disability or handicap (disability and handicap now called “activity limitation” and “participation restrictions,” respectively, in the International Classification of Functioning Disability and Health; World Health Organisation 2001). A clinical measure of listening effort could also inform interventions that redress these important aspects of hearing disability.

In research settings, various purported measures of listening effort have been used including: (1) self-report such as National Aeronautics and Space Administration (NASA) Task Load Index (Hart & Staveland 1988), (2) behavioral such as reaction time, for example, Houben et al. (2013) and dual task, for example, Desjardins and Doherty (2013), and (3) physiological such as galvanic skin response, for example, Mackersie et al. (2015), electroencephalographic measures, for example, Petersen et al. (2015), pupillometric indices, for example, Zekveld et al. (2011), and functional near-infrared spectroscopy, for example, Wijayasiri et al. (2017). However, it is not clear if these measures tap into the same construct and this may explain, at least in part, why the different measures rarely correlate with each other (McGarrigle et al. 2014). Multiple measures of listening effort have generally not been obtained simultaneously while the participant performs a listening task, making it difficult to make a direct comparison between the measures. The reliability of alternative listening effort measures must be established before they could be considered for use in research or clinical settings (Koo & Li 2016). Unreliable measures are unlikely to correlate strongly with each other, even if they index the same construct.

## MEASURES OF LISTENING EFFORT AND FATIGUE

McGarrigle et al. (2014) and Pichora-Fuller et al. (2016) provide a detailed discussion of the self-report, behavioral, and physiological measures that have been used in listening effort/fatigue research. Ohlenforst et al. (2017a) also provide a systematic review of studies that investigated the effect of hearing impairment or the effect of hearing aid amplification on listening effort. Table 1 provides a summary of the measures and their main advantages and disadvantages.

**TABLE 1.**
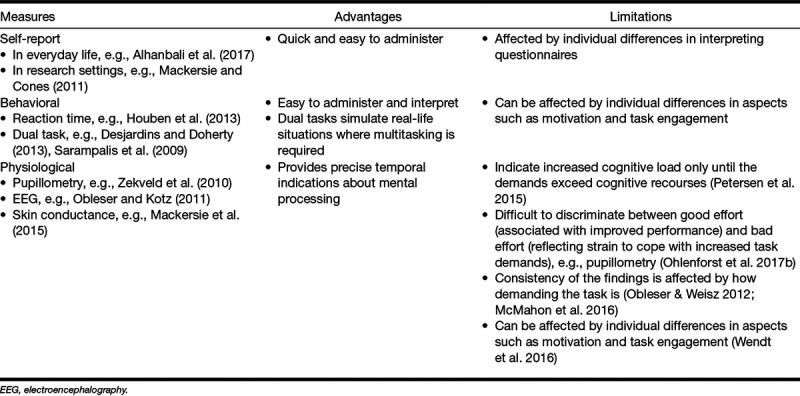
The advantages and limitations of using self-report, behavioral, and physiological measures of listening effort

Inconsistencies between different measures of listening effort and fatigue have been reported including disagreement between different (1) measures, (2) participant groups, and (3) studies that used the same measure to test similar groups of participants but used different listening tasks. The variability in the testing methods used across different studies (including speech material, participants, listening conditions) complicates the ability to directly compare their results (Ohlenforst et al. 2017a). Therefore, it is not clear if these inconsistencies are because the measures assess different processes or because some measures are unreliable or lack sensitivity. Further discussion on the inconsistencies reported in the literature is provided in the following section.

### Inconsistent Associations Between Measures

Inconsistent correlations between self-report, behavioral, and physiological measures of listening effort have been reported. Variability in experimental methods and the aspects of cognitive/emotional demands that may be assessed by different measures could have contributed to these inconsistent findings. Some studies have reported associations between self-report, behavioral, and physiological measures. For example, associations between self-reported arousal and pupil size in response to emotionally evocative visual and auditory stimuli (Burley et al. 2017); a relation between prestimulus pupil diameter and electroencephalography (EEG) activity in an auditory oddball paradigm (Hong et al. 2014); an association between pupil size and event related potentials activity in monolingual and bilingual toddlers (Kuipers & Thierry 2013). However, other studies focused on listening effort reported no association between putative self-report, behavioral, and physiological measures of listening effort. For instance, increased pupil size during a listening task has previously been interpreted as reflecting increased effort, for example, Zekveld et al. (2011). However, in some instances (Wendt et al. 2016), increased pupil size was found to be associated with decreased self-reported effort and improved task performance. Therefore, larger pupil dilation was sometimes considered an indication of higher levels of attentional focus and vigilance that does not necessarily translate to perceived effort and strain (Wendt et al. 2016).

Meaningful correlations between different physiological measures of listening effort are often lacking. For example, McMahon et al. (2016) reported no correlation between alpha power and pupil size when participants performed a task that involved listening to noise-vocoded sentences. The authors suggested that the lack of correlation might be due to different neurophysiological or attentional networks that modulate the activity of the physiological processes indexed by the different measures. Similarly, it was suggested that the often reported nonsignificant correlations between self-report and behavioral/physiological measures of listening effort may be because self-report and behavioral/physiological measures assess different aspects of listening effort/fatigue (Mackersie et al. 2015).

### Inconsistent Patterns Between Different Groups of Participants Tested Using the Same Measure

Greater EEG alpha band (8 to 12 Hz) power was reported to index increased listening demands (Obleser et al. 2012; Dimitrijevic et al. 2017). However, patterns of change in alpha band power are inconsistent between studies. Petersen et al. (2015) reported increased alpha power in participants with normal hearing or mild hearing loss as the listening conditions became more challenging and the demands of the listening task increased. However, decreased alpha power was identified in participants with moderate hearing loss in the more challenging listening conditions. Petersen et al. suggested that participants with moderate hearing loss had exerted maximal cognitive effort in the challenging listening conditions so that further increases in alpha power were not possible. The authors suggested that the decrease in alpha power is likely a result of participants “running out” of cognitive resources.

The pattern of change in peak pupil size was also found to vary between groups of participants with normal hearing and participants with hearing impairment as the demands of a listening task increased (Ohlenforst et al. 2017b). According to Ohlenforst et al. (2017b), different patterns of change in peak pupil size across participants with normal hearing and participants with hearing impairment suggest that they allocate cognitive effort differently depending on task demands.

### Inconsistent Findings Between Studies That Used the Same Measures But Different Listening Material

Inconsistent findings have been reported for the same physiological measure of listening effort in different studies that recruited similar groups of participants but used different listening material, for example, Obleser and Weisz (2012) and McMahon et al. (2016). While Obleser and Weisz reported decreased alpha power suppression (i.e., increased alpha power) when listening to words with less spectrotemporal detail, McMahon et al. reported increased alpha power as the intelligibility of the sentences presented to the participants increased.

The different listening materials used across the studies might explain the contradictory findings. Obleser and Weisz (2012) presented participants with noise-vocoded single words while McMahon et al. (2016) presented participants with noise-vocoded sentences. Kahneman’s (1973) model of attention suggests that listening is often an “automatic” process in ideal listening conditions. However, degradation of inputs limits the ability to map inputs to automatic representations in the memory. Processing of sentences might be associated with increased limitations on the ability to automatically process speech inputs when trying to establish the relation between the different items in the sentence. A nonmonotonic relation exists between task demands and listening effort (Ohlenforst et al. 2017b). Therefore, the difference in the listening demands associated with processing different speech materials complicates the ability to compare the results of different studies.

In summary, a variety of self-report, behavioral, and physiological measures of “listening effort” have been used in research studies. Although all measures have been interpreted in terms of “listening effort,” measures do not always agree well with each other, across participant groups, or between studies. The first explanation might be that measures are unreliable. Unreliable measures are unlikely to correlate with each other. The second explanation might be the inconsistencies in the listening tasks used across studies. Measures might correlate with each other if the same listening task is used. The third explanation might be that the various self-report, behavioral, and physiological measures may encompass different concepts that are related to listening effort, including arousal, attention, stress, and perceived difficulty (Pichora-Fuller et al. 2016). The various measures might also assess different processes or neural mechanisms involved in effortful listening, such as verbal working memory and attention-based performance monitoring (Peelle 2017). If there are multiple dimensions of “listening effort,” then multiple measures may be required for the assessment of listening effort. One final explanation might be that different measures tap into underlying phenomena that are independent of the concept of listening effort. The use of the various measures of listening effort was based on models and theories that provided links between increased listening demands and the potential measures. However, the absence of a gold standard for the assessment of listening effort limits the ability to confirm that the different measures relate to the concept of listening effort.

## AIMS

Multiple potential measures of listening effort were recorded simultaneously during a listening task that involved listening to digits in background noise in a large group of adult participants with a range of hearing levels. Measures included (1) two self-report measures (NASA Task Load Index and the Visual Analog Scale of Fatigue [VAS-F]) and (2) three physiological measures (pupillometry, skin conductance, and EEG). Other potential indicators of listening effort included performance on a speech-in-noise task and participants’ hearing level. The rationale for using each of the measures is provided below:

- Participants’ perception of listening difficulties should be the main interest in hearing rehabilitation. Therefore, the inclusion of a self-report measure in the design of this study was considered essential and theoretically sound.
- The use of EEG alpha power in the assessment of listening effort is based on the inhibition theory that suggests that increased alpha power is likely to occur in tasks requiring the retention of learned information or the suppression of irrelevant inputs (Klimesch et al. 2007). Therefore, changes in alpha activity during a retention period where participants are required to memorize learned information was used as an index of listening effort (Obleser et al. 2012). Increased alpha power while listening to speech in background noise was considered a potential indicator of effortful listening associated with the suppression of background noise (McMahon et al. 2016). Alpha power in the baseline period was also considered a potential indicator of listening effort. According to Klimesch et al. (2007), increased baseline alpha activity is an indicator of pretask cortical engagement that predicts improved task performance. Wang et al. (2018) have also suggested that the activity during a baseline period (reflected by pupil size during the baseline) might be related to task engagement. Including a predictor of task performance was motivated by recent reports suggesting that the accuracy of task performance can influence the experience of listening effort (Pichora-Fuller et al. 2016).
- Increased alertness results in increased pupil size (Kahneman 1973). Therefore, pupillometry has been traditionally considered an index of increased levels of alertness that might occur in demanding listening conditions (McGarrigle et al. 2014). On other occasions, increased pupil size has been considered an indication of increased task engagement associated with motivation and successful performance (Kuchinsky et al. 2014). Pupillometry provides an online method for momentary assessment of the changes in the ongoing neural activity during the performance of demanding tasks performance (Peelle 2017).
- Skin conductance provides an indication about the activity in the autonomic system. Activity in the sympathetic nervous system increases in demanding conditions to prepare the body to expend increased energy during the “fight or flight” response (McArdle et al. 2006). On this basis, skin conductance was considered a candidate measure of listening effort associated with listening to speech in demanding conditions (Mackersie & Cones 2011).
- Performance on a speech in noise task was considered a candidate measure of listening effort. Evidence suggests that performance on a speech task correlates with self-reported listening effort (Alhanbali et al. 2018). The accuracy of performance on a listening task can influence listening effort, for example, successful task performance can motivate further exertion of listening effort and vice versa. Therefore, performance accuracy might provide an indication about the experience of listening effort in individual participants (Ohlenforst et al. 2017b).
- Participants’ hearing level was also considered a candidate indicator of listening effort. Despite the lack of correlation with self-reported effort (Alhanbali et al. 2017), the pattern of change in a number of listening effort measures (such as pupillometry and EEG alpha power) depends on participants’ hearing level (Petersen et al. 2015; Ohlenforst et al. 2017b).

The first aim of this study was to assess the reliability of the measures by testing a subgroup of participants on two separate occasions. The second aim was to assess the correlation between the different measures. The final aim was to use factor analysis (FA) to identify whether purported, reliable measures of listening effort assess similar or different underlying factor(s).

## METHODS

### Participants

Participants were native English speakers recruited from the database of three UK National Health Service audiology departments and via flyers posted around the University of Manchester campus and through social groups. A total of 141 participants took part in the study. The data of 25 participants were not included in the FA due to problems in the pupil or in the EEG data as will be described later. Therefore, data for 116 participants were included in the FA. The age range of the participants whose data were included in the FA was 55 to 85 years (mean: 70, SD: 8), with 50.3% males. Participants’ hearing thresholds were established using pure-tone audiometry. Hearing thresholds in the better ear of individual participants’ ranged from 10 to 77 dB HL over the frequencies 500, 1000, 2000, and 4000 Hz (mean: 33, SD: 16.7). Participants with hearing level ≤30 dB HL at any individual frequency from 500, 1000, 2000, and 4000 Hz (n: 37, age: 55 to 84 years) were classified as having good hearing. Participants whose hearing level fell outside the “good” category were classified as having mild hearing impairment (mean: 31 to 40 dB HL; n: 42, age: 68 to 83 years), moderate hearing impairment (mean: 41 to 70 dB HL; n: 29, age: 55 to 83 years), or severe hearing impairment (mean: 71 to 95 dB HL; n: 8, age: 61 to 83 years). These classifications resemble those defined by the British Society of Audiology, though with a higher minimum threshold for the mild category. Seventy participants were prescribed hearing aids by the National Health Service. All participants used behind-the-ear hearing aids with nonlinear amplification fit according to the National Acoustics Labs, Non-Linear, version 1 prescription target. Self-reported use was reported as “most of the day” for a minimum duration of 6 months. Participants performed the listening task with the hearing aid settings as used in everyday life. The purpose of using everyday hearing aid settings was to measure listening effort in a cross-section of current hearing aid users, as was done by Alhanbali et al. (2018). Therefore, we did not directly measure real ear gain to confirm audibility or if the hearing aids met the prescription target.

The sample size was determined on the basis of providing adequate statistical power to support a FA, that is, a minimum of 5 to 10 participants per variable (Field 2009), with a minimum of 100 participants in total (Floyd & Widaman 1995). The study was reviewed and approved by the National Research Ethics Services of South Central-Hampshire A, Research Ethics Committee reference: 15/SC/0113.

## MATERIALS

### Listening Tasks

The speech material was monosyllabic digits “1” to “9” from the Whispered Voice test (McShefferty et al. 2013) recording of a male speaker. Bisyllabic number “7” was not included. The masker was unmodulated background noise. The noise started 5 sec before the onset of the first digit and ended 1 sec after the last digit had ended. Five seconds of noise is usually sufficient for the automatic noise reduction function in hearing aids to activate*. The signal to noise ratio (SNR) was determined using a sequence of three digits.

The listening task was performed in a sound-treated booth. The speech material was presented at a fixed level of 65 dB(A). Speech and background noise were both presented via loudspeakers at ±45° azimuth. Participants were seated facing a computer monitor. The height of the chair was adjusted to achieve the most comfortable setting for the participants with the head position supported using a chin rest.

In an attempt to equalize intelligibility across participants, the SNR required for each participant to report 71% correct identification of sequences of 3 digits was established before performing the main listening task, where listening effort was recorded using the different measures. Refer to Alhanbali et al. (2018) for details about establishing 71% criterion performance. In summary, the individualized SNR for each participant was established (for sequences of three digits) using a two-down, one-up, with a 2-dB step size adaptive procedure. This replicates approaches taken in previous studies (Mackersie et al. 2015; Petersen et al. 2015). The mean SNR for criterion performance of 71% correct was −4 dB (SD: 5 dB). Unlike the three-digit sequence used to determine individualized SNRs, the main study used sequences of six digits to maximize the cognitive demands of the task. Within each sequence of six digits, each digit was not repeated more than twice (e.g., 2 6 8 5 1 8). The minimum number of unique digits within each sequence was five, that is, only one digit could be presented twice within each sequence. The main listening task was a modified version of the Sternberg paradigm (Sternberg 1966) in which participants had to memorize speech material presented during a stimulus-free retention period based on similar paradigms described by Obleser et al. (2012) and Petersen et al. (2015). The listening task was programmed using SR research Experiment Builder software (SR Research version 1.10.1630, Mississauga, ON, Canada). Participants with hearing impairment performed the task with their hearing aids on.

Before the main listening task, participants watched a documentary for 10 min (the baseline period) to acclimatize to the experimental setting and to obtain baseline values for skin conductance (see Physiological Measures section). The task started by presenting participants with the message “press ENTER when you are ready.” The word “Listen” then appeared on the screen and 5 sec of unmodulated noise followed by the first sequence of six digits in noise were presented. A 3-sec retention period followed, during which participants had to fixate on a cross while mentally rehearsing the digits. A digit then appeared on the screen and an audible pure tone was presented to alert the participant to respond. Using a button box with “Yes” and “No” labels, participants responded with “Yes” if the digit on the screen was one of the digits they heard and with “No” if it was not. After responding, there was a recovery period of silence for 4 sec before the start of a new trial to allow measures to return to baseline. Before the start of the main listening task, participants performed 10 practice trials of six digit sequences at their individualized SNR. The total number of experimental trials was 50. The overall duration of the listening task was around 15 min.

**Fig. 1.**
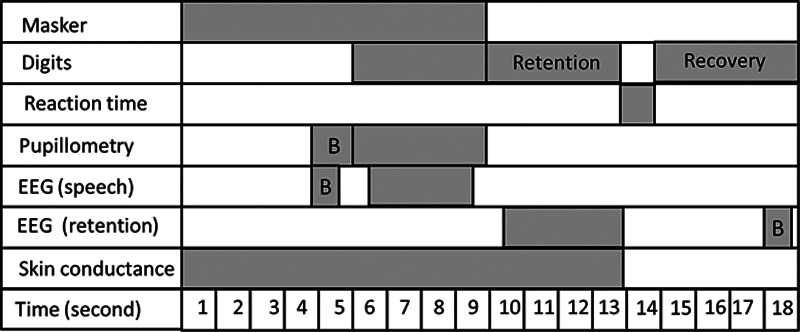
An outline of the sequence of events in each trial and the time periods used when analyzing the data for each measure. Retention: the period during which participants memorized the digits; recovery: the period before the start of a new trial. B indicates baseline period; EEG, electroencephalography.

Figure 1 provides an outline of the sequence of events in each trial and the time periods used when analyzing the data obtained from the different measures, as will be discussed later.

### Reliability of the Measures

A subgroup of 30 participants performed a retest 1 wk after the first test session. According to Koo and Li (2016), a minimum of 30 samples (participants) are required to provide enough power for reliability testing. Both testing sessions were performed at the same time of day.

### Listening Effort and Fatigue Measures

#### Self-Report Scales

NASA Task Load Index and the VAS-F (Lee et al. 1991) were used for measuring self-reported listening effort and fatigue, respectively. NASA Task Load Index is a standardized measure for the assessment of perceived demands during task performance. The NASA Task Load Index consists of six items including mental demand, physical demand, temporal demand, perceived performance, effort, and frustration. After performing the main listening task, participants provided responses on a 20-step scale ranging from low demand to high demand for each dimension. The score of each item was converted to a percentage. The total score was calculated based on the mean score of the items used.

The VAS-F consists of 2 subscales which are fatigue (12 items; e.g., fatigued, tired, and exhausted) and energy (6 items; e.g., active, energetic, and efficient). For each question, participants had to respond by choosing one number on a scale with two distinct points ranging from 0 to 10. For the fatigue items, larger numbers indicate more fatigue, while for the energy items larger numbers indicate more energy. The scales of the different items were converted so that they changed in the same direction. The total score was calculated based on the mean score of the items used. Participants completed the VAS-F before and after performing the main listening task. Final scores were based on the difference in mean VAS-F before and after performing the listening task. Although the duration of the listening task was only around 15 min, the development of fatigue was expected to occur due to the repetitive nature of the task that required participants to provide prompt responses (Hockey 2013).

#### Behavioral Measure

Reaction time was used as the behavioral measure of listening effort. The time between the response prompt and participants’ response (button press) was recorded in milliseconds for both the correct and the incorrect responses and then averaged across all trials for each participant. Reaction time information was exported through the SR research Experiment Builder software. Reaction times were obtained after the “stimulus-free period” (Fig. 1). Ideally, measurement of reaction time should commence immediately after the stimulus ends. In the current paradigm, the inclusion of a retention period was essential for the EEG and pupillometry data, as will be discussed in the sections later. As a result, the retention period may mean that the data are contaminated by the addition of a memory recall component. For this reason, we did not include reaction time in the analysis. In addition, performance differences in the listening task between the groups (see below) complicate the ability to draw conclusions about listening effort based on the reaction time measure. Performance in the listening task should ideally be equalized across participants when using reaction time as a measure of listening effort (Houben et al. 2013; Strand et al. 2018).

#### Physiological Measures

##### Pupillometry:

###### Pupillometry recording:

Pupil sizes were measured using an Eyelink 1000 with a sampling rate of 1000 Hz. The eye tracker was connected to the same PC that was used to present the listening task. The desktop mount of the Eyelink 1000 was used and the eye tracker was placed just below the lower edge of the computer monitor. Pupil size was measured based on the number of pixels in the pupil image captured by the camera which ranged from 100 to 1000 units with a precision of 1 unit corresponding to 0.01 to 5 mm pupil diameter. Pupil size was changed into mm by calculating the number of pixels in an artificial pupil with a known size.

The camera of the eye tracker was calibrated by asking participants to fixate on a black circle that periodically appeared at one of nine different coordinate positions on the computer monitor. Based on the luminance adjustment procedures reported in Zekveld et al. (2010), room lighting and screen brightness were adjusted for each participant to avoid floor/ceiling effects in pupil size. For each participant, pupil size was recorded in a bright (room brightness at 263 lux and screen brightness at 123 cd/m^2^) and a dark setting (room brightness at 0.28 lux and screen brightness at 0.0019 cd/m^2^). Room lighting and screen brightness were then adjusted to achieve a pupil size that was in the middle range of the bright and the dark setting. The pupil size of the right eye was measured for all participants.

###### Pupillometry data preprocessing:

In each trial, the pupil data included in the analysis ranged from the start of the speech stimulus and until the end of the 4-sec retention period. Consequently, each epoch included the duration of the speech stimulus presentation plus the 3-sec retention period (Fig. 1). The 3-sec retention period was included in the analysis because of the lag of the peak pupil response that was observed in previous research (Piquado et al. 2010; Zekveld et al. 2010).

Pupil data were analyzed based on previous studies (Zekveld et al. 2010, 2011) using MATLAB (MathWorks Inc., version R2015a, MA) scripts. Missing data points due to eye blinks were removed from the analysis. Trials with more than 15% of missing data points between the start of the baseline period to the end of the retention period were removed from the analysis (Zekveld et al. 2011; Ohlenforst et al. 2017b). Linear interpolation using data points before and after the blink was applied to replace missing data points. Data were smoothed using 5-point moving average to remove any high-frequency artifacts. The mean number of trials lost for each participant was 5 (SD: 2). A total of 15 participants had more than 10 trials rejected due to problems, such as drooping eyelids or diagnosed lazy eye, and were thus excluded from the analysis.

###### Pupillometry data analysis:

Once artifactual trials had been removed, the remaining trials were used to obtain two pupil outcome measures: (1) peak pupil dilation amplitude, and (2) mean pupil dilation amplitude. Mean pupil size during the 1 sec that preceded the presentation of the speech stimulus was used as a baseline (Fig. 1). Peak and mean pupil dilation were calculated relative to baseline, that is, peak and mean pupil dilation were subtracted from mean pupil size during baseline. A single overall mean and peak pupil dilation for each participant was based on the mean and peak pupil size from the average of all the accepted trials.

##### EEG:

###### EEG recording:

EEG was recorded using a Nexus-10 physiological recording system with the BioTrace software (Mind Media neuro and biofeedback system). EEG was sampled at 256 Hz with no online filtering. Increased alpha activity associated with increased listening effort has mainly been observed over the parietal lobe (Obleser & Weisz 2012; Obleser et al. 2012). Seven silver/silver chloride electrodes with a sintered surface were used. Three positive electrodes were therefore placed over parietal scalp regions to capture task-related alpha activity: Pz, P3, and P4 based on the international 10 to 20 system (Homan et al. 1987). One additional positive electrode was placed at Cz because each of the two positive electrodes were connected to one reference electrode. The two reference electrodes were placed on both ear lobes. One additional ground electrode was placed at the forehead. Before placing the EEG electrodes using conductive paste, the skin was prepared using an abrasive gel. Electrode impedance was kept below 5 ohms.

###### EEG data preprocessing:

EEG data were processed using EEGlab tool box (Delorme & Makeig 2004). The first 0.5 sec of any predetermined time periods (baseline/noise/speech/retention) were excluded from the analysis so as to avoid any stimulus onset or offset activity. Data were filtered with a low-pass cut-off of 45 Hz and a high-pass cut-off of 5 Hz to remove artifacts resulting from eye blinks using EEG lab (Cohen 2014). Filtered data were then epoched to include 2-sec prestimulus onset and 1 sec after the end of the trial and including the retention period when analyzing alpha power during the retention period (Fig. 1). When analyzing alpha power during speech presentation, epochs commenced with the start of the noise (5 sec of noise before the start of speech). Trials containing artifacts, including blinks, saccadic eye movements, or electromyography activity, were removed from further analysis. Participants’ data with more than 20% rejected trials were not included in the analysis (Cohen 2014). The mean number of trials lost for each participant was 7 (SD: 3). A total of 10 participants had more than 10 trials contaminated with artifacts and were excluded from further analysis.

###### EEG time-frequency analyses:

**Fig. 2.**
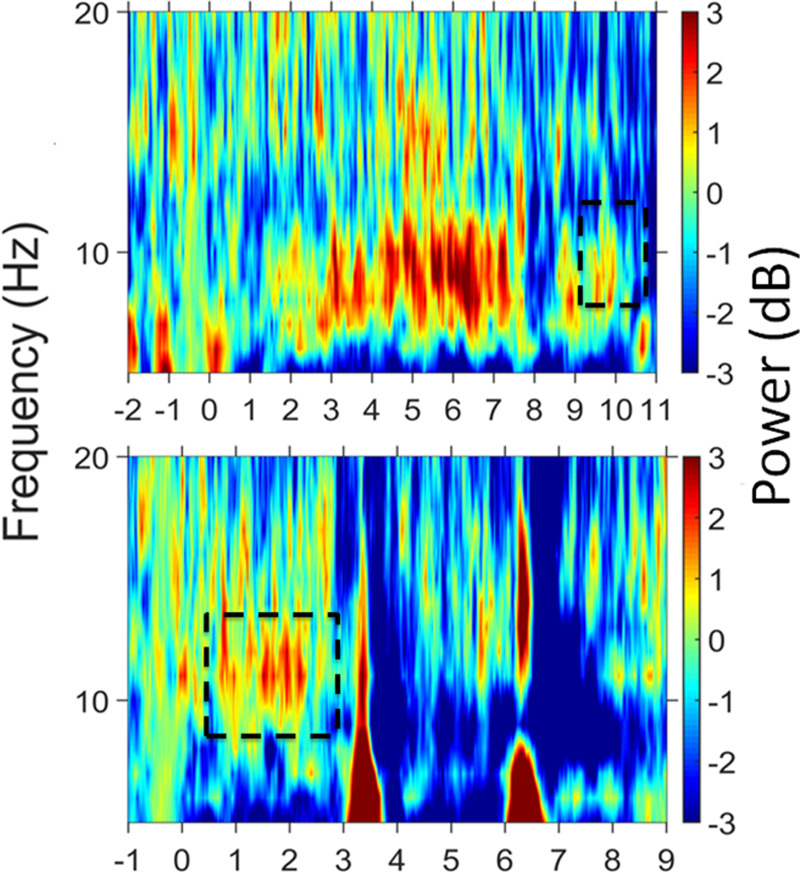
Mean change in alpha power across participants and trials. The temperature scale represents changes in event-related band power in decibels (dB). The top panel shows changes in alpha activity during the retention period relative to baseline alpha activity in the recovery period, that is, before the noise is presented. The bottom panel shows changes in alpha activity during the speech presentation period relative to alpha activity during the last second of unmodulated noise, that is, the period of noise alone that preceded the presentation of the first spoken digit. Dashed boxes represent the time periods of increased alpha activity (n =116). The X-axis represents time.

Time-frequency decomposition using Morlet wavelet convolution was applied to the data. Complex wavelet convolution was performed to quantify changes in event-related band power (ERBP; Nourski et al. 2009) over the time periods outlined in Figure 1 (−2 to 13 sec around the onset of a trial). ERBP for the retention period (Fig. 2, top panel) was estimated for each center frequency from 5 to 20 Hz in 1 Hz steps. Based on previous research (Petersen et al. 2015), power estimates during the retention period were calculated relative to power estimates during the prestimulus baseline period (−0.6 to −0.1 sec before stimulus onset). Based on Dimitrijevic et al. (2017) and McMahon et al. (2016), alpha power estimates were also calculated during the speech presentation period (Fig. 2, bottom panel). Alpha power changes during the speech presentation period were contrasted with a different baseline that was defined during the presentation of noise alone (−0.6 to −0.1 sec before the speech onset). This approach ensured that any increase in alpha power during the speech presentation period resulted from the response to the presentation of speech and not merely the response to the presentation of a sound (Dimitrijevic et al. 2017). Power estimates during the prestimulus baseline period (−0.6 to −0.1 sec of the stimulus onset) were calculated and included in the FA to determine whether prestimulus alpha predicted task performance (Klimesch et al. 2007).

The alpha ERBP was quantified for each individual participant in the center frequencies ranging from 8 to 13 Hz using EEGlab tool box. To do so, trial data were convoluted with a family of 3 Morlet waves (default setting of EEGlab). Measures of alpha power were obtained using the power calculation function in EEGlab (spectopo). Alpha power was calculated during the prestimulus baseline (−0.6 to −0.1 sec) and the retention period (9.5 to 12 sec into the trial). Alpha power was also calculated during the noise baseline period (−0.6 to −0.1 sec before the speech onset) and during the presentation of the speech (5.5 to 8.5 sec into the trial). For each center frequency within the alpha band (8 to 13 Hz) and each time point, power estimates were obtained by calculating the logarithm of the mean power during (1) the retention period over the mean power during the baseline period or (2) the mean power during the speech period over the mean power during the noise period (Fig. 1). Alpha power was then averaged across the center frequencies within the alpha band and time period of interest. Alpha power was calculated for each trial, averaged across trials for each participant.

To visualize a time-frequency representation of the data (Fig. 2), customized MATLAB scripts developed by Nourski et al. (2009) were used. Time-frequency decomposition using Morlet wavelet convolution (2πƒ_0_ σ = 7) (Petersen et al. 2015) was applied to the data averaged across all participants. The entire filtered frequency range, that is, 5 to 45 Hz is not presented in Figure 2 to allow a better visualization of changes in alpha power (8 to 13 Hz).

##### Skin Conductance

Recordings of skin conductance and EEG were performed simultaneously via separate channels in the Nexus-10. Skin conductance was sampled at 32 Hz. Two silver/silver chloride electrodes were attached to the index and the middle finger of the participant’s nondominant hand. Participants were instructed to keep their hand facing palm-up to minimize artifacts resulting from hand movement or any pressure applied on the electrodes.

Skin conductance data were extracted through the Biotrace software. The epoch of each trial commenced from the start of the stimulus and terminated at the end of the retention period. We did not include the 4-sec recovery period in the skin conductance analysis as participants did not do any mental task during that period.

To account for the individual differences in baseline skin conductance, mean skin conductance for each participant across all trials was corrected to baseline. Pilot testing indicated that it took around 3 min for the skin conductance values to settle. As a result, average skin conductance value in the 7 min that preceded task performance (while watching the documentary) was used as a baseline. Mean skin conductance across trials was subtracted from mean skin conductance in the baseline period. The value resulting from the subtraction was then divided by mean skin conductance in the baseline period.

### Statistical Analysis

The data were not normally distributed and were therefore summarized using median and interquartile ranges (IQR), and analysis involved nonparametric tests. Test–retest reliability was assessed using Spearman’s correlation coefficient (consistency of the results across the testing sessions) and interclass correlation coefficient (ICC; test–retest reliability). ICC estimates and 95% confidence interval were calculated based on an absolute agreement one-way random effects; ICC1 based on Shrout and Fleiss (1979). ICC1 is sensitive to differences in means between the observations and is a measure of absolute agreement. Each session for each participant can be considered a separate condition due to differences in aspects such as electrode placement or how alert the participant is on the day of testing. Therefore, every session can be regarded as being conducted by a separate “rater” or “judge” suggesting that ICC1 is likely the most appropriate to use for these data (Shrout & Fleiss 1979). The relations between the different variables were investigated using Spearman’s correlation coefficient. The correlation between each of the different variables and age was also investigated.

The suitability of the data for a FA was investigated using Kaiser–Meyer–Olkin measure of sampling adequacy (KMO) test and Bartlett’s test of sphericity (Field 2009). FA included only the measures that were shown to have good retest reliability (see later). Factors were identified based on eigenvalues greater than one (Field 2009). It was initially expected that the different factors would correlate with each other. Therefore, oblique rotation was considered appropriate for identifying how measures load into distinct factors (Field 2009). Multiple parameters of EEG and pupillometry were included in the FA because these might tap into independent aspect of increased listening effort. For example, increased alpha activity during the retention period was considered an indication of increased demands on working memory (Petersen et al. 2015) whereas increased alpha activity during the speech presentation period was considered an indication of suppression of background noise (McMahon et al. 2016). Furthermore, measures of EEG alpha during the baseline period may be predictive of task performance (Klimesch et al. 2007).

## RESULTS

### Test–Retest Reliability

**Fig. 3.**
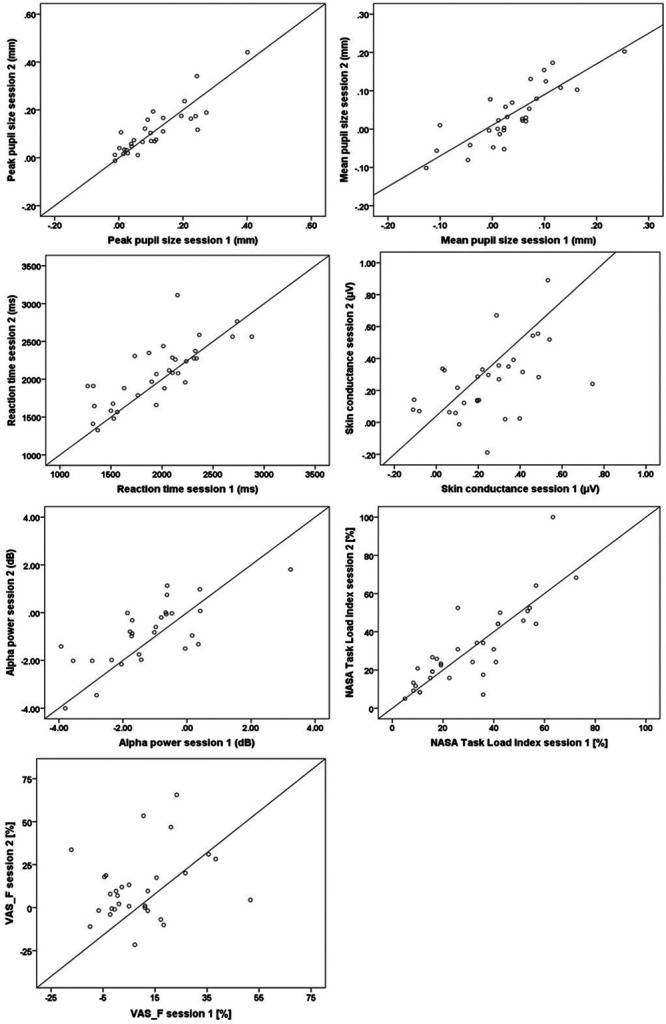
Associations between the test (x axis) and retest (y axis) data (n = 30). NASA indicates National Aeronautics and Space Administration; VAS_F, Visual Analogue Scale of Fatigue.

Figure 3 shows the relationship between the test and retest results. Spearman’s correlation coefficients and ICC with 95% confidence interval for the different measures are summarized in Table 2. Spearman’s correlation coefficients indicated excellent consistency across the testing sessions for all measures except for skin conductance, which was moderately consistent, and VAS-F which had poor consistency. Pupillometry had excellent reliability, EEG (alpha power) had good reliability, reaction time had good reliability, skin conductance had fair reliability, NASA Task Load Index had excellent reliability, and VAS-F had poor reliability based on the ICC classification suggested by Cicchetti (1994). Skin conductance and VAS-F were not included in the FA due to relatively poor test–retest reliability.

**TABLE 2.**
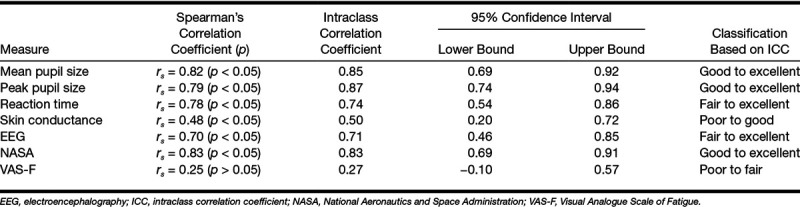
Correlation coefficients between the test and the retest sessions of the different measures and results of ICC calculation with confidence intervals

### Descriptive Statistics

SNR was adjusted for each participant to identify 71% of the digits triplets presented. Mean SNR was −9 dB (SD: 2) for participants with good hearing, −3 dB (SD: 3) for participants with mild hearing loss, −1 dB (SD: 5) for participants with moderate hearing loss, and +4 dB (SD: 4) for participants with severe hearing loss. However, actual performance in the main listening task was better than 71%. This is probably because participants were not required to repeat every digit they heard in the main listening task. Mean performance on the main listening task was 93% (SD: 4) for participants with good hearing, 89% (SD: 7) for participants with mild hearing loss, 87% (SD: 6) for participants with moderate hearing loss, and 82% (SD: 6) for participants with severe hearing loss. Participants’ performance in the main listening task was high for all groups (range, 82 to 93%). Results of one-way analysis of variance revealed a significant difference in mean performance between groups [*F*(3,93) = 10.1; *p* < 0.05]. Pairwise comparison with Bonferroni correction suggested that participants with good hearing performed better than the three groups with hearing loss (*p* < 0.013). However, there was no significant difference in the mean performance of the three groups with hearing loss.

The median score and IQR for the NASA Task Load Index were 34.16% (IQR: 26.25). For VAS-F, the values were 6.50% (IQR: 17.96). For reaction time, the values were 1945.86 millisec (IQR: 540.71) and for skin conductance, 0.25 µS (IQR: 0.30).

### Pupillometry

**Fig. 4.**
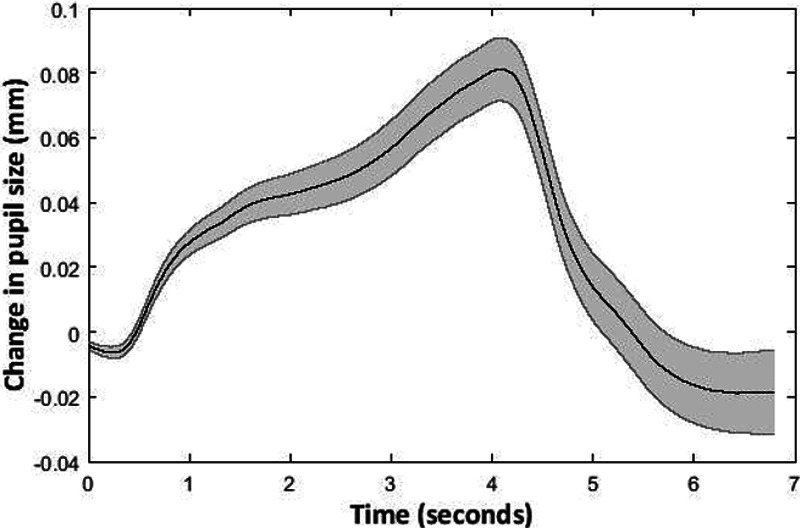
Mean change in pupil size relative to baseline across participants and trials. The black line represents mean change in pupil size across participants and trials (y) axis. The shaded gray area represents ±1 SE. (n = 116). Time in seconds (0 to 4 sec: speech presentation period, 4 to 7 sec: retention period) is shown on the x axis.

Figure 4 shows mean change in pupil size across all participants (n = 116) and trials. Pupil size increased significantly relative to baseline as participants attended to the speech, and reached a peak toward the end of the 3-sec speech stimulus. Median mean pupil size across participants was 0.02 mm, IQR = 0.08. Median peak pupil size was 0.11 mm, IQR = 0.12.

### Electroencephalography

Figure 2 shows the mean ERBP (Nourski et al. 2009) across participants (n = 116) and trials. The top panel represents mean ERBP during the retention period relative to baseline during the recovery period. The bottom panel represents mean ERBP during the presentation of the digits in noise relative to baseline during the presentation of the noise only. Changes in ERBP are represented by the temperature scale which ranges from −5 to 5 dB. Figure 2 suggests an increase in alpha activity toward the end of the retention period and an increase in alpha activity during speech presentation (8 to 13 Hz; highlighted by black dashed box). A Wilcoxon rank test was used to establish whether alpha power during the retention period and during the speech presentation period significantly increased compared with their respective baselines. Increased alpha activity was identified during the speech presentation period only (0.5 to 4 sec); (*z* = −2.30; *p* = 0.05). Median baseline-corrected alpha power during speech presentation across participants was 0.17 dB, IQR = 1.99. Median baseline-corrected alpha power during retention was −0.95 dB, IQR = 1.50. Median baseline alpha power across participants was 2.24 μv, IQR = 3.21.

## CORRELATIONS AND FA

Some weak to strong correlations were identified between the variables (Table 3). No corrections for multiple comparisons were applied. Age was weakly correlated with SNR (*r* = 0.29; *p* < 0.05) and not correlated with any other measure. Therefore, age was not included in the FA. FA involved nine variables:

**TABLE 3.**
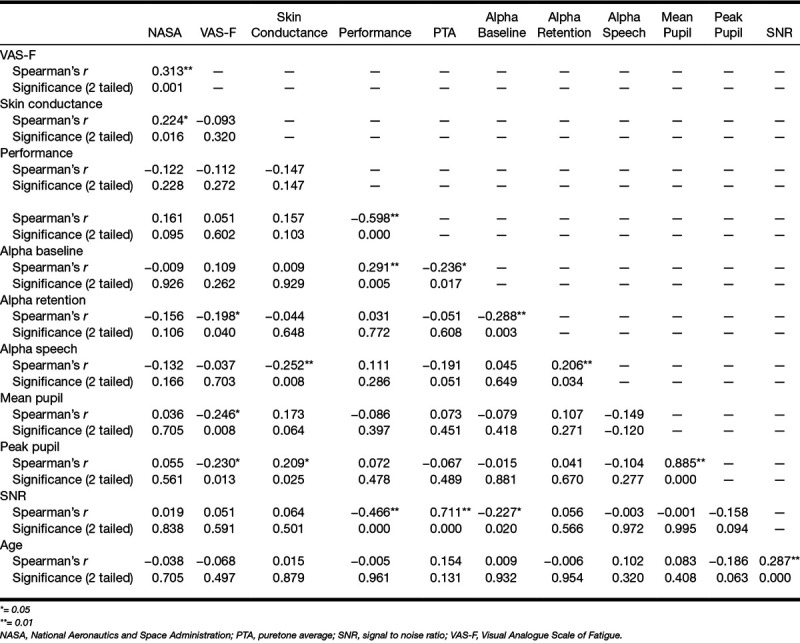
Correlation coefficients between the measures

1. NASA Task Load Index
2. SNR required to identify 71% of the digit triplets presented
3. Mean pupil size
4. Peak pupil size
5. EEG alpha during baseline period
6. EEG alpha during retention period
7. EEG alpha during speech presentation
8. hearing level and
9. performance accuracy in the main listening task that required correct recall of a random digit from a sequence of six.

**Fig. 5.**
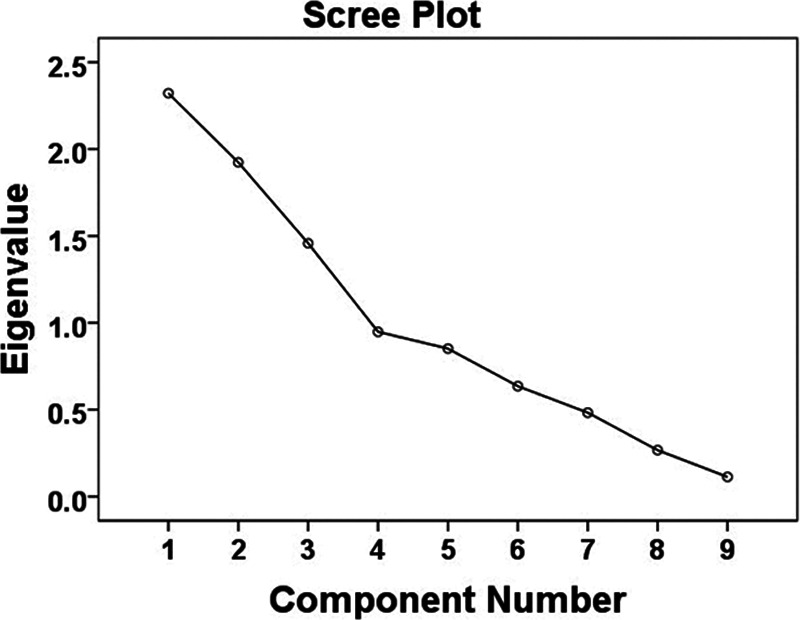
Scree plot and extracted eigenvalues.

Results of a KMO test (0.57) indicated the adequacy of the sample size for a FA (Field 2009). According to Field (2009), KMO values below 0.50 are unacceptable for a FA; therefore, the result of the KMO test (0.57) indicated a low but adequate sample size for a FA (Field 2009). Bartlett’s test of sphericity *X*^2^(36) = 247.17, *p* < 0.001, indicated that correlations between the variables were sufficient for a FA. The determinant of the correlation matrix in the FA was 0.05. FA yielded 3 factors with eigenvalues >1 that explained about 63% of the total variance (factor 1: performance accuracy in the main listening task, hearing level, SNR, and baseline alpha power; factor 2: mean pupil size and peak pupil size; factor 3: self-reported effort, alpha power during the speech presentation and the retention periods). However, the inflection point on the scree plot (Fig. 5) suggested that extracting four factors is more appropriate. The amount of variance explained increases from 63 to 74% when 4 factors are extracted instead of 3 factors (Table 4).

**TABLE 4.**
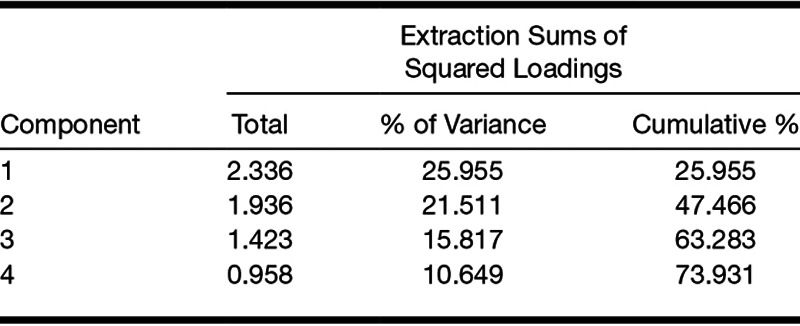
Percentage of the variance explained by each factor

Table 5 provides details of the loading of each variable onto the different factors. To facilitate the interpretation of the data, low loadings of less than 0.30 are not shown (Field 2009). Both structure and pattern matrices yielded similar results (with the exception that the pattern matrix suggests that baseline alpha power loads into factors 1 only), so only the structure matrix is reported here. Oblique rotation resulted in performance accuracy in the main listening task, hearing level, baseline alpha power, and SNR loading into factor 1. Mean pupil size and peak pupil size loaded into factor 2. Alpha power during the speech presentation and the retention periods loaded into factor 3. Self-reported effort and baseline alpha power loaded into factor 4. Table 6 shows the correlation matrix for the four factors identified. The weak correlations suggest that the factors identified are independent, that is, orthogonal (Field 2009).

**TABLE 5.**
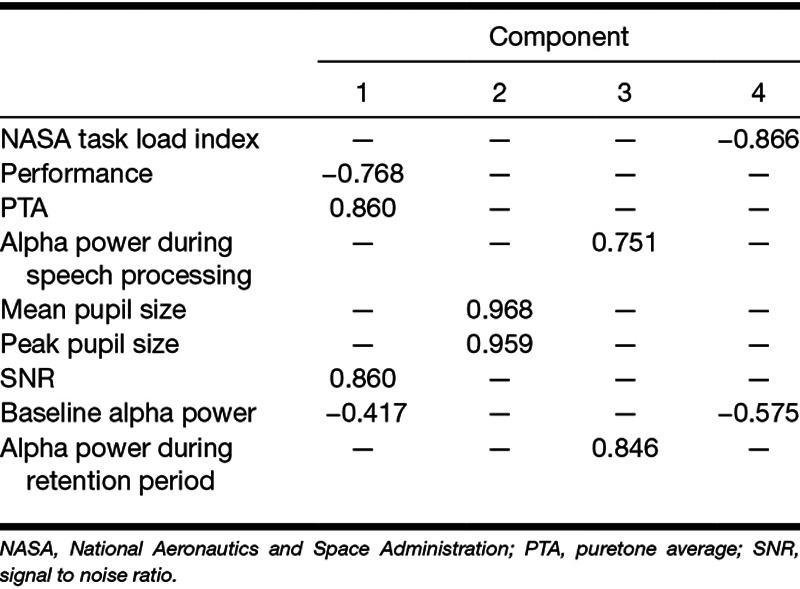
Structure matrix: factor loadings of the variables to each factor

**TABLE 6.**
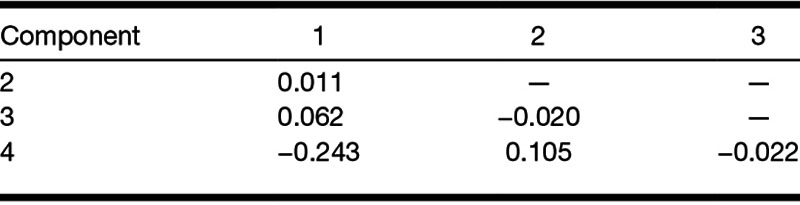
Matrix of correlation coefficients between the factors

## DISCUSSION

### Test–Retest Reliability

To our knowledge, the present study is the first to report the reliability of different measures of listening effort. All measures except skin conductance and VAS-F had good-to-excellent reliability. Although results of a Wilcoxon rank test suggested the absence of a significant difference in mean skin conductance across both test sessions, it was only moderately reliable. Skin conductance is more sensitive to emotional factors than the other measures used in this study (Hogervorst et al. 2014) and this might explain the higher variability for this measure. Results of pilot testing indicated that skin conductance has low temporal resolution; because this measure required 3 min to stabilize. Low temporal resolution might have also contributed to the low reliability of the measure. Future study should consider data analysis approaches that take into account the low temporal resolution of changes in skin conductance.

VAS-F also showed poor test–retest reliability. VAS-F scores were based on the difference (after − before) in participants’ rating of fatigue. On inspection of the individual measures before and after task performance, pretask fatigue ratings were unreliable (ICC: 0.26), while post-task fatigue ratings showed good reliability (ICC: 0.83). Poor reliability of the baseline fatigue measures could have been influenced by multiple factors that are difficult to control across participants, for example, factors associated with various aspects of daily life before the commencement of the test sessions. In contrast, fatigue rating after the task was most likely based on task performance and thus was more likely to be reliable. This suggests that a direct measure of change in fatigue (i.e., having participants rate change in fatigue after performing a listening task) may be a better measure than deriving the difference between two states (i.e., comparing self-reported fatigue before and after task performance). This is consistent with Gatehouse (1999) who demonstrated the improved discriminatory capability of using a single change measure.

### Multidimensionality of Listening Effort Measures

Despite good reliability of the measures, they were only weakly correlated with each other. The use of different measures was motivated by theories and models which suggested their sensitivity to increased listening demands. Therefore, weak correlations between reliable measures suggest that they tap into different underlying dimensions. Agreement on what the concept of listening effort encompasses has not yet been reached within the discipline of audiology. It remains unclear if listening effort is a single concept, or if it is an umbrella term for multiple phenomena. Matthen (2016) suggested that a one-factor model is unlikely to explain the experience of listening effort. The different factors identified in this study support the argument that listening effort involves multiple dimensions.

Multidimensional cognitive psychological models of attention might provide a relevant framework for understanding the multidimensionality of listening effort. Kahneman’s (1973) model of attention suggests that effort is the consequence of mismatch between the cognitive demands of a task and the supply of cognitive resources. Pichora-Fuller et al. (2016) suggested that Kahneman’s model of attention is relevant to effortful processing of degraded speech because it suggests that the presence of a perceptual deficit is a direct cause of increased effort. Kahneman’s model suggests that multiple dimensions control the allocation of cognitive resources for task performance, such as the levels of arousal and the evaluation of task demands with respect to capacity. Therefore, Pichora-Fuller et al. hypothesized that multiple factors influence the experience of effortful listening. In an elaboration on Kahneman’s model of attention, Pichora-Fuller et al. suggested that measures of listening effort might tap to the multidimensional attention-related outputs of Kahneman’s model (including cognitive-behavioral changes, changes in brain activity, changes in autonomic nervous system activity, and in self-report measures) in their Framework for Understanding Effortful Listening (FUEL) model. The multidimensional attention-related outputs described in the FUEL model are consistent with the finding that different potential measures of listening effort tap into different underlying dimensions.

FA suggested that there was considerable redundancy with the nine variables grouping into four underlying factors. Hearing level, SNR, EEG activity during the baseline period, and performance accuracy in the main listening task loaded into factor 1: better hearing was associated with decreased SNR, better task performance, and increased EEG activity during the baseline period. Peak and mean pupil size loaded into factor 2 in the same direction. EEG alpha power during speech presentation and during the retention period loaded in the same direction into factor 3. EEG activity during the baseline period and self-reported effort loaded in an opposite direction into factor 4: increased EEG activity during the baseline period was associated with decreased self-reported effort. The pattern of correlations between the measures might have been influenced by the single, individualized SNR at which the task was performed. Future study should consider investigating the pattern of correlations across different levels of SNR and for different listening tasks.

### Implications of the Multidimensionality of Listening Effort

The finding that the nine alternative measures of “listening effort” mapped onto four separate factors is consistent with previous research findings that did not identify a correlation between alternative physiological measures (McMahon et al. 2016), physiological and self-report measures (Wendt et al. 2016), and physiological measures and hearing level (Petersen et al. 2015). The identification of these separate factors might imply that, rather than looking for a gold standard of how to measure listening effort, we should be more precise in our descriptions and interpretations of what is measured in a single experiment.

The distinction between the terms “listening effort,” “cognitive effort,” and “mental effort” is under-specified in the literature. “Cognitive effort” and “mental effort” may be more general than “listening effort,” which is specific to processing auditory inputs. However, these terms have been used interchangeably in previous research to reflect the demands associated with listening, for example, Zekveld et al. (2011) and Koelewijn et al. (2012). A clear distinction between the terms might help to determine if the dimensions assessed by potential measures of listening effort are indicators of generic mental effort or whether they are specific to changes associated with listening effort. For example, different measures of “mental load” (pupil size, skin conductance, and heart rate) may differ in terms of peak response latency and in relation to task demands (Kahneman et al. 1969). Such differences support the multidimensionality of “mental load” or “listening effort,” as described in the present study.

Listening is multidimensional and the listening task employed here (correct recall of a digit presented in noise) likely required auditory-cognitive interactions. According to the FUEL model (Pichora-Fuller et al. 2016), cognitive domains involved in listening include working memory, attention, and speed of processing. Each of the factors identified in this study may capture a different dimension of listening effort. Therefore, the multidimensionality of the measures identified in this study support previous suggestions by Pichora-Fuller et al. (2016) that inferences about listening effort should be considered in light of different components that could be revealed through self-report, behavioral, and physiological measures.

Establishing the underlying dimensions of the different factors and how they are related to the concept of listening effort might not be straightforward and is beyond the scope of this study. To establish the underlying dimensions of the measures, future study might consider the following approaches: (1) conducting a detailed literature review with the aim of investigating which concepts different measures have been related to in previous cognitive psychology research, (2) establishing the construct validity of the measures by investigating how they correlate with measures that are valid indices of their hypothesized underlying concepts, and (3) investigating whether the measures systematically change according to task/listening demands or individual characteristics, as would be predicted by an appropriate model (e.g., FUEL, Pichora-Fuller et al. 2016).

## LIMITATIONS

An attempt to equalize intelligibility across participants was carried out using a task that involved presenting participants with digit triplets. However, when obtaining recordings using purported measures of listening effort, participants performed a listening task that required correct recall of a random digit from a sequence of six. It is possible that estimating the SNR required for repeating three digits was not optimal for a six-digit recognition memory task. This might have resulted in differences in performance across participants. However, it should be noted that participants’ performance in the main listening task was high for all groups (range, 82 to 93%). Future study should consider using a similar listening task when establishing a certain criterion performance and when obtaining recordings of listening effort.

The SNR at which the participants performed the listening task may have also been unrealistically challenging compared with real-life situations. The SNRs at which participants were tested actually represent listening situations that individuals would usually avoid (Smeds et al. 2015). The purpose of choosing challenging SNRs in lab settings is usually to avoid ceiling effects. However, this might not be essential if measures of listening effort are sensitive to changes in performance at SNRs that are more representative of real-life situations. For example, Krueger et al. (2017) reported that a measure of self-reported listening effort revealed that the use of hearing aids or noise reduction algorithm results in decreased listening effort when participants listened to speech in favorable listening conditions. Future study should consider obtaining simultaneous recordings while participants perform a listening task at noise levels that are representative of real-life situations. Evidence suggests that performance accuracy can influence the results obtained using different listening effort measures. Therefore, performing the listening task at more favorable SNRs would also allow an in-depth investigation of the effect of task performance on listening effort. The use of multi-talker background noise may have been more representative of real-life situations compared with unmodulated noise. Greater listening effort has been reported in the presence of a single-talker “informational” masker compared with unmodulated noise (Koelewijn et al. 2012). Future study should consider the aforementioned factors when obtaining simultaneous recordings of the different measures. Consideration of these methodological factors might eliminate any effect they might have had on the factors identified, for example, participants’ performance in the listening task was considered to have influenced the loading direction of a number of the measures.

Asking participants to memorize spoken digits may not have been sufficiently demanding to be sensitive to differences in effort. A task requiring manipulation of speech information might tap into aspects of everyday listening that might be more cognitively demanding and more sensitive to changes in listening effort. For instance, Rabbitt (1990) did not identify an effect of hearing impairment on participants’ ability to correctly repeat words; however, hearing impairment reduced the participants’ ability to correctly recall the words. The shift from the auditory to the visual modality when presenting the response prompt might have unintentionally increased the cognitive demands associated with task performance compared with providing information through the auditory modality only. Future study might need to consider presenting the speech inputs and the response prompt through a single modality.

The relatively low KMO value suggests that the interpretation of the FA results should be treated with caution and justifies the need for independent replication of this study. Finally, the improved discriminatory capability of using a direct measure of change (e.g., in self-reported fatigue) is an important psychometric principle to be borne in mind when designing self-report measures and evaluating change.

## CONCLUSIONS

This is one of the very few studies directly comparing simultaneously recorded multimodal measures purported to index listening effort. The measures used in this study tap into independent dimensions. Establishing the underlying dimensions assessed by each of the measures needs to be considered in the future study. Measures should not be used interchangeably as each of them appears to tap into an independent aspect of listening demands. The present study suggests the importance of careful and in-depth consideration of the aspect of increased listening demands of interest before choosing an appropriate measure. As such, the findings of the present study have widespread implications for both research and clinical practice.

## ACKNOWLEDGMENTS

We thank Dr. Andrew Dimitrijevic for assistance in developing the research protocol. We also thank Dr. Mohammad Sobuh, Dr. Emanuele Perugia, and Dr. Garreth Prendergast for their assistance in the data analysis.

## Footnotes

* Resound noise tracker II, White paper.
